# Chronic psychological stress alters gene expression in rat colon epithelial cells promoting chromatin remodeling, barrier dysfunction and inflammation

**DOI:** 10.7717/peerj.13287

**Published:** 2022-04-29

**Authors:** John W. Wiley, Gerald A. Higgins, Shuangsong Hong

**Affiliations:** 1 Department of Internal Medicine, University of Michigan - Ann Arbor, Ann Arbor, MI, United States of America; 2 Department of Computational Medicine and Bioinformatics, University of Michigan - Ann Arbor, Ann Arbor, MI, United States of America

**Keywords:** Chronic stress, Visceral hyperalgesia, Colon epithelial cells, RNA-sequencing, Tight junctions, Cytokines, Epigenetic regulation, Chromatin remodeling

## Abstract

Chronic stress is commonly associated with enhanced abdominal pain (visceral hypersensitivity), but the cellular mechanisms underlying how chronic stress induces visceral hypersensitivity are poorly understood. In this study, we examined changes in gene expression in colon epithelial cells from a rat model using RNA-sequencing to examine stress-induced changes to the transcriptome. Following chronic stress, the most significantly up-regulated genes included *Atg16l1*, *Coq10b*, *Dcaf13*, *Nat2*, *Ptbp2*, *Rras2*, *Spink4* and down-regulated genes including *Abat*, *Cited2*, *Cnnm2, Dab2ip*, *Plekhm1*, *Scd2*, and *Tab2*. The primary altered biological processes revealed by network enrichment analysis were inflammation/immune response, tissue morphogenesis and development, and nucleosome/chromatin assembly. The most significantly down-regulated process was the digestive system development/function, whereas the most significantly up-regulated processes were inflammatory response, organismal injury, and chromatin remodeling mediated by H3K9 methylation. Furthermore, a subpopulation of stressed rats demonstrated very significantly altered gene expression and transcript isoforms, enriched for the differential expression of genes involved in the inflammatory response, including upregulation of cytokine and chemokine receptor gene expression coupled with downregulation of epithelial adherens and tight junction mRNAs. In summary, these findings support that chronic stress is associated with increased levels of cytokines and chemokines, their downstream signaling pathways coupled to dysregulation of intestinal cell development and function. Epigenetic regulation of chromatin remodeling likely plays a prominent role in this process. Results also suggest that super enhancers play a primary role in chronic stress-associated intestinal barrier dysfunction.

## Introduction

Intestinal barrier dysfunction has been implicated in several common clinical disorders including mood disorders, obesity and non-alcohol fatty-liver disease (NAFLD), diabetes mellitus, and enhanced abdominal pain (visceral hypersensitivity) associated with irritable bowel syndrome (IBS). IBS is a common functional gastrointestinal disorder affecting 5–10% of the general population worldwide. Despite its relatively high prevalence, the cellular pathophysiology of visceral hypersensitivity is poorly understood but most likely multifactorial and involves both central and peripheral mechansims (Chey, Shah & DuPont, 2020; Mearin et al., 2016; Chang et al., 2018; Johnson et al., 2020). Recent evidence support that chronic psychosocial stress likely plays a significant role in bowel dysmotility and visceral hypersensitivity (Faresjö et al., 2007; Nicholl et al., 2008), which is influenced by the type of stressor, its intensity and duration (Imbe, Iwai-Liao & Senba, 2006).

The central (CNS) and peripheral pathways through which chronic stress influences gastrointestinal disorders have not been fully resolved, but intestinal epithelial barrier dysfunction associated with increased paracellular passage of macromolecular species has been proposed to be a mechanistic link. Intestinal barrier dysfunction has been observed in a subset of patients with IBS and other functional gastrointestinal disorders (Martinez et al., 2013; Vanheel et al., 2014; Stewart, Pratt-Phillips & Gonzalez, 2017). Emerging evidence support that enhanced intestinal epithelial cell paracellular permeability correlates with the severity of visceral pain in animal models following psychological stress (Creekmore et al., 2018; Dunlop et al., 2006; Phua et al., 2015). Changes in permeability in gastrointestinal disorders have been linked to decreased expression of adherens and tight junction proteins including e-cadherin, claudin(s), zonula-occludens 1 (ZO-1), and occludin in intestinal epithelial cells. However, the cellular and molecular pathways that regulate chronic stress-induced down-regulation in epithelial cell junction gene expression are poorly understood.

A variety of rodent models have been employed to assess the pathophysiology of visceral pain and identify prospective biomarkers as potential diagnostic or therapeutic targets for treatment of gastrointestinal disorders such as IBS. While no rodent model completely recapitulates the complex pathophysiology of IBS in the human, several animal models exhibit visceral hypersensitivity and have been employed for mechanistically-focused studies and to screen potential novel therapeutics (Johnson et al., 2020). The psychosocial chronic, intermittent water avoidance stress (WAS) model is one of the most commonly used rodent models that displays down-regulation of intestinal epithelial tight junction proteins, increased paracellular permeability, visceral hypersensitivity and increased fecal output, and is thought to better represent the psychological stress and anxiety that triggers IBS symptoms in humans (Bradesi et al., 2009; Hong et al., 2009; Larauche et al., 2010; Tran et al., 2013; Zheng et al., 2013). Little is known about the cellular and molecular pathophysiology underlying how chronic stress induces intestinal barrier dysfunction and visceral hypersensitivity. In this study, we employed the chronic WAS rat model to profile the transcriptomes of intestinal epithelial cells following chronic stress and screen the molecular/cellular pathways underlying chronic stress-induced visceral hypersensitivity. Our findings will help elucidate the molecular and cellular characterizations of chronic stress-associated changes in intestinal epithelial cell transcriptome which is a significant contributing factor to the pathophysiology of visceral hypersensitivity and IBS.

## Materials and Methods

### Animals and water avoidance stress

This study was approved by the University of Michigan Committee on Use and Care of Animals according to National Institutes of Health guidelines (IACUC protocol PRO00005713). All experiments were carried out in accordance with the guidelines of the Institutional Animal Care and Use Committee. Male Sprague-Dawley rats weighting 160–180 g were obtained from Charles River Laboratories (Wilmington, MA, USA). Animals were randomly grouped and housed in the animal facility that was maintained at 22 °C with an automatic 12-h light/dark cycle. The animals received a standard laboratory diet and were adapted for 7 days in the animal facility before subject to chronic psychological water avoidance stress (WAS) procedure (Creekmore et al., 2018; Zheng et al., 2013). In the morning the adapted rats were placed on a glass platform in the middle of a tank filled with water (22 °C) to one cm below the height of the platform and maintained for 1 h daily for 10 consecutive days. Sham control animals were put into the same tank without water for 1 h every day for 10 days. Body weight and fecal output during the 1-h WAS or sham stress for each rat were recorded. Total RNAs were extracted for sequencing and bioinformatics analysis as shown in the scheme (Fig. S1).

### Isolation of rat colon epithelial cells

On the following day after the completion of 10-day WAS or sham stress, rats were sacrificed by CO_2_ inhalation and distal colon segments (2–6  cm from anus) were removed and perfused with ice-cold DPBS (without Ca^2+^ and Mg^2+^). The lumen side was inverted to the outside for incubation in DPBS containing 4 mM EDTA and 0.5 mM DTT for 15 min at 4 °C. Colon crypts and epithelial cells were detached and collected by centrifuge at 50 × g for 2 min at 4 °C. After a brief wash with ice-cold DPBS, the enriched colon epithelial cells were frozen in liquid nitrogen until analysis.

### RNA extraction

Total RNA was extracted from thecolon epithelial cells using the Trizol reagent (Life Technologies, Carlsbad, CA, USA) following manufacturer’s protocol as previously described (Hong, Zheng & Wiley, 2015). RNA integrity and quality were assessed by Bioanalyzer 2100 and gel electrophoresis. The RNA concentration and purity were determined at A260 nm and A280 nm wavelengths using a NanoDrop Spectrophotometer (Thermo Fisher Scientific, Waltham, MA, USA). The quality of the total RNA that was used for cDNA library preparation met the following standards: OD260/280 = 1.8∼2.2, OD260/230 = 2.0∼2.2, RIN ≥ 8.0.

### Library preparation and Illumina HiSeq Sequencing

Preparation of cDNA library was conducted using the Illumina library construction kit and total RNA sequencing was performed on a HiSeq 4000 sequencing platform (Illumina, San Diego, California, USA) at the University of Michigan Advanced Genomics Core. Briefly, ribosomal RNA was removed using Epicentre Ribo-Zero Gold Kits (Epicentre, Madison, WI, USA) according to the manufacturer’s protocol. The libraries were assessed for quality using Agilent High Sensitivity DNA kit and Agilent 2100 Bioanalyzer (Agilent Technologies, Santa Clara, CA, USA). cDNA libraries were single-end sequenced with read length of 50 bases. The library reads of greater than 30 million were generated for each individual library.

### Analysis of differentially expressed genes

Sequencing raw reads were pre-processed for quality assessment, trimming, quality filtering and size selection using the FASTQC toolkit to generate quality plots for all read libraries. The sequencing data were then mapped to the *Rattus norvegicus* reference genome (*Ensembl* Rnor_6.0 version 99) using STAR (Dobin et al., 2013) v2.7 to generate sorted BAM and unsorted transcriptome BAM files. The quality metrics of the mapped data were collected using the Picard tools suite (v2.22). The raw read counts obtained directly by STAR were quantified at the gene and exon-intron-junction levels. Annotation of genes were based on the corresponding *Ensembl* annotation files of *Rattus Norvegicus*. In the present study the pipeline employed Sleuth (Pimentel et al., 2017), DESeq2 (Love, Huber & Anders, 2014) and edgeR (Robinson, McCarthy & Smyth, 2010) statistical methods from the R (v3.6) Bioconductor package to call differentially expressed genes (DEGs). Based on a negative binomial data model (Love, Huber & Anders, 2014; Anders & Huber, 2010), statistical comparisons were made using calculation of false discovery rate (FDR) with the commonly used threshold in differential RNA-Seq analysis of FDR <0.01. To ensure data normalization and removal of bias based on variables such as transcript length, several methods were used, including conditional quantile normalization and preprocessing using a low count filter.

### Analysis of differential transcript usage (DTU) and differential exon usage

Differential transcript usage was analyzed by Relative Abundance of Transcripts (*RATs*) (Froussios et al., 2019) that identifies DTU at both gene and transcript levels. For direct comparison with differential gene expression, the transcript abundance of each sample was quantified by Salmon (Patro et al., 2017) v1.21 from the transcriptome BAM file generated by STAR. DTU for each sample was then identified and analyzed by *RATs*.

### Pathway analysis of differentially expressed transcripts

Different applications were used to determine enriched biological processes including Gene Ontology (GO), Kyoto Encyclopedia of Genes and Genomes (KEGG), gene set enrichment analysis (GSEA) (Subramanian et al., 2005) and Reactome (Yu & He, 2016) pathway databases. Identification of canonical pathways, upstream regulators, and network reconstruction was performed using a bioinformatics workflow that included Ingenuity Pathway Analysis (IPA) (Kramer et al., 2014) as described in a previous publication (Higgins et al., 2017).

### Annotation with disease risk SNPs from genome wide association studies (GWAS) in humans

Human analogs of both the significantly up-regulated and down-regulated genes were evaluated for significant associations of single nucleotide polymorphisms (SNPs) in the NHGRI-EBI GWAS database (Buniello et al., 2019).

## Results

### Changes in gene expression in rat colon epithelial cells following chronic psychological stress

Transcriptomic analysis was performed using RNA-seq comparing differential gene expression in the colon epithelial cells between chronic WAS rats and sham controls. Alignment statistics showed that the percentage of mapping to the coding region was significantly decreased in the WAS rats. The percentage of mapping to the untranslated region (UTR) was significantly increased. No significant differences were observed for the percentages of mapping to the intron and intergenic regions between control and WAS groups (Table S1).

**Figure 1:**
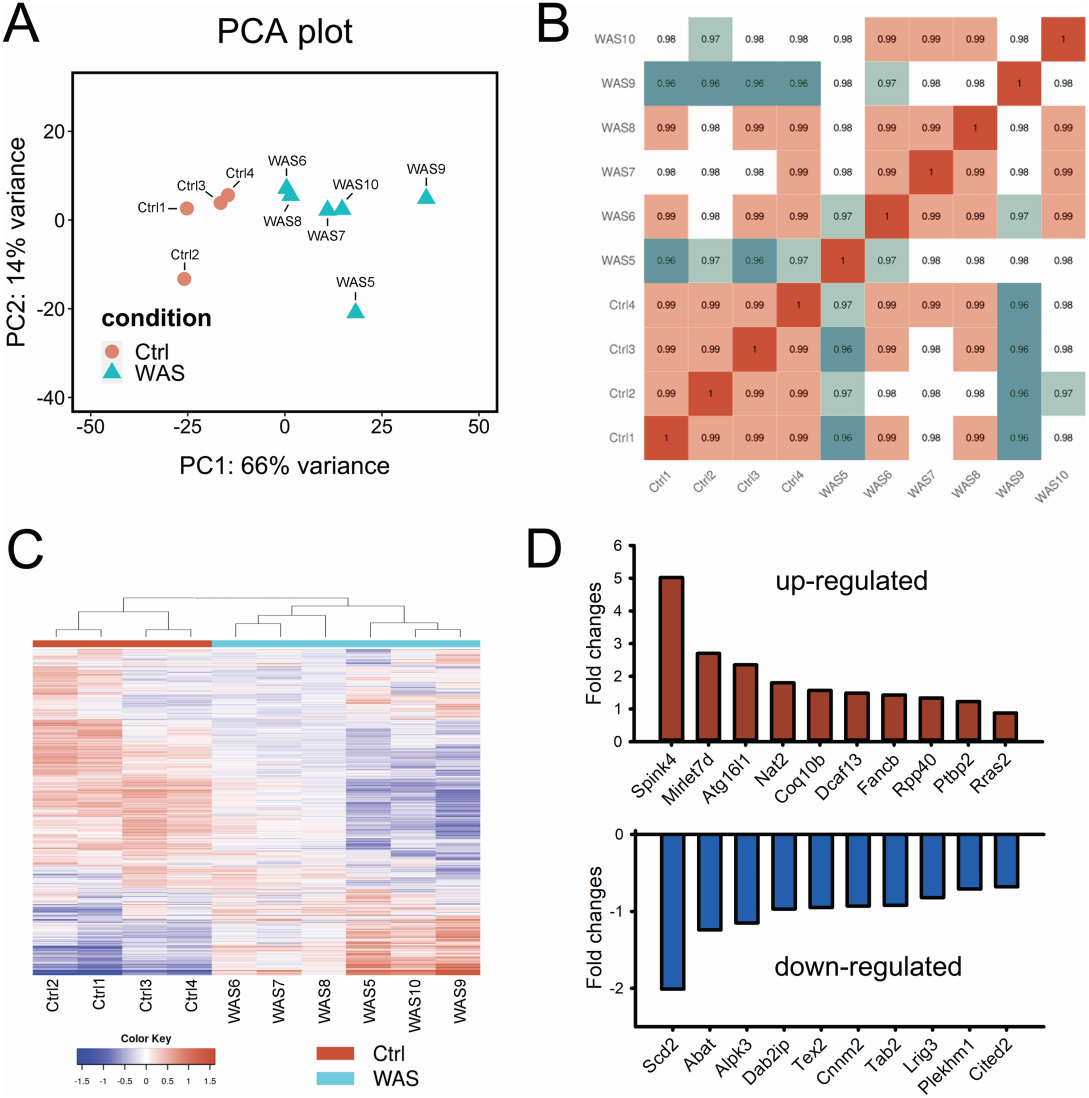
Chronic stress caused significant changes in gene expression profile in rat colon epithelial cells. (A) PCA plot showed the similarity and difference between each sample and between control and WAS rats. (B) Heatmap of gene expression correlation between each tested sample. (C) Hierarchical cluster analysis of differently expressed mRNAs. The color scale indicates log_2_(CPM+1) and intensity increases from blue to red, which indicates down- and up-regulation, respectively. (D) Top 10 upregulated (red) and down-regulated (blue) genes in chronic WAS rats compared to controls. Adjusted (adj.) *P* values < 1.27E-06.

Principal component analysis (PCA) demonstrated differences between the control samples and the WAS samples (Fig. 1A). Results of statistical analysis visualized by a heatmap of inter-sample correlation showed high correlations of the samples with only minor variation among the WAS group (Fig. 1B). Analysis of differential gene expression (DEG) showed that 1826 genes were significantly up-regulated and 890 genes were significantly down-regulated in WAS samples compared to controls (adj. *P* < 0.01). Hierarchical clustering of the top 2000 DEGs (fold change > 1.5 and adj. *P* < 0.01 by FDR) showed a significant change in the WAS samples compared to the controls, including the emergence of a distinct subpopulation of transcripts following chronic psychological stress (Fig. 1C). As shown in Fig. 1D, the top 10 most significantly up-regulated transcripts were *Spink4*, *Mirlet7d*, *Atg16l1*, *Nat2*, *Coq10b*, *Dcaf13*, *Fancb*, *Rpp40*, *Ptbp2* and *Rras2*, while the top 10 most significantly down-regulated genes were *Scd2*, *Abat*, *Alpk3*, *Dab2ip*, *Tex2*, *Cnnm2*, *Tab2*, *Lrig3*, *Plekhm1* and *Cited2* in WAS rats compared to controls using the DESeq2 method (details shown in Table S2). In contrast, the D-GEX deep learning method yielded two additional top upregulated genes (*Scarna5* and *Nod2*). The magnitude of WAS-induced changes in differential expression was larger for both the upregulated and downregulated gene sets (Table S3), and at the extremes, were consistent with amplification of gene transcription by super-enhancers. (Wei et al., 2020) The deep learning method that we employed did not detect microRNA *Mirlet7d* (Table S3).

### DEGs annotated with disease risk SNPs from genome wide association studies (GWAS)

To better characterize the functional genomics of these DEGs, we examined the human analogs of both the significantly up-regulated and down-regulated genes for significant associations of the single nucleotide polymorphisms (SNPs) contained within their human counterparts in the NHGRI-EBI and UK Biobank GWAS databases. Emphasis was placed on diseases and traits in humans that have previously been characterized as resulting from stress-related etiology, including depression, anorexia nervosa, anxiety and related traits, inflammatory disorders, and gastrointestinal disease (Table S4). SNPs within 8 of the 23 top up-regulated and down-regulated genes harbor gene variants that are significantly associated in humans with Crohn’s disease, chronic inflammatory disease, inflammatory bowel disease, ulcerative colitis, depression, anxiety and related traits, anorexia nervosa, asthma, mouth ulcers and related traits (Table S4).

### GSEA and GO enrichment analyses of DEGs in chronically stressed rat epithelial cells

**Figure 2:**
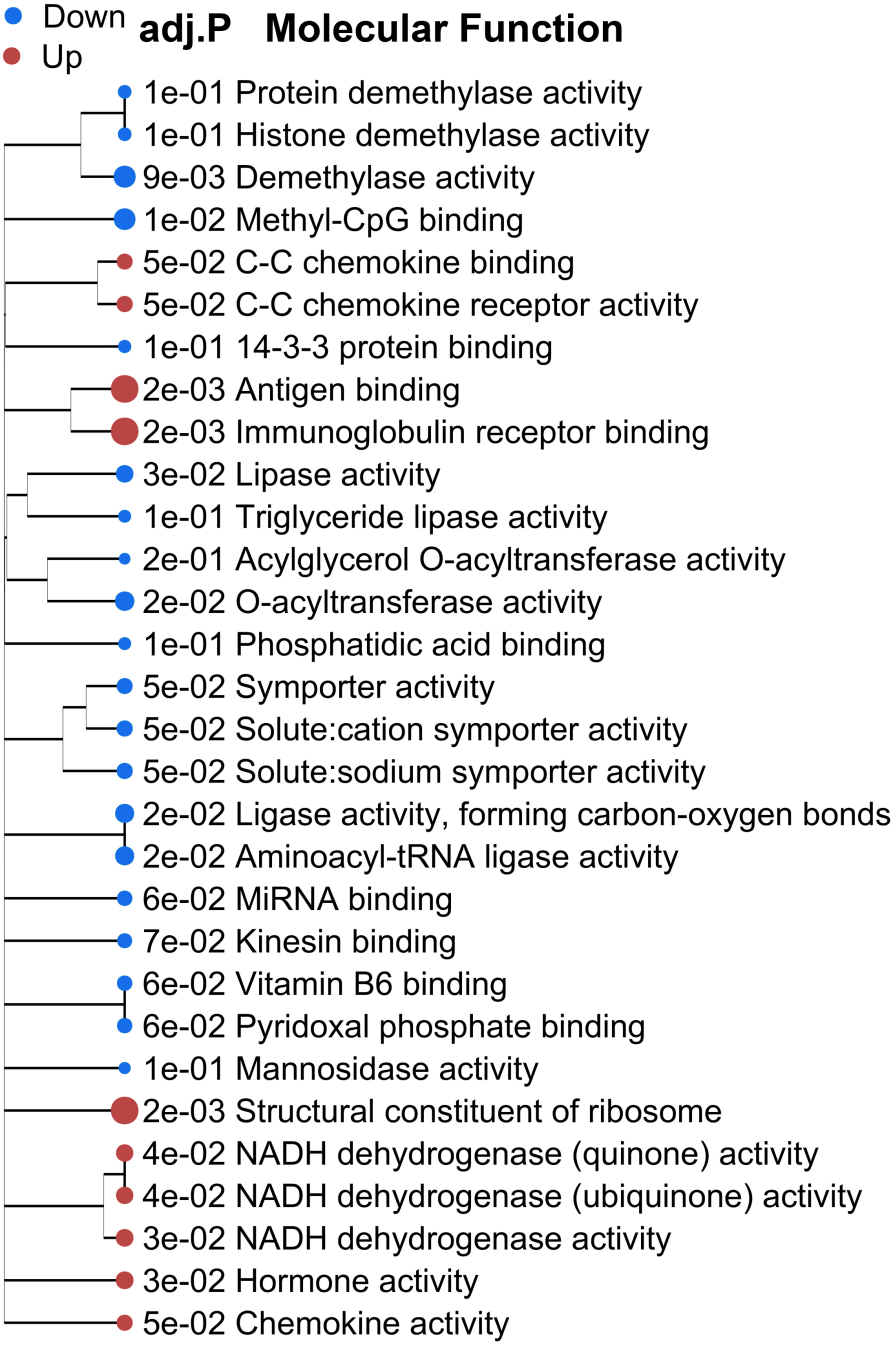
Pathway view of significantly altered molecular functions in GO terms analyzed by preranked GSEA method in chronic WAS rats compared to controls. Red: upregulated; blue: down-regulated. Adj. *P* < 0.05 by FDR.

The top 2,000 DEGs were then processed for GSEA (preranked) and GO enrichment analyses. GSEA analysis showed significantly down-regulated molecular functions such as histone demethylase and lipids activities in WAS rats, while antigen binding, hormone and chemokine activities were upregulated (Fig. 2). Go enrichment using K-means clustering (Yu & He, 2016) also revealed gene clusters related to the immune system process (adj. *P* < 6.20E-24), anatomical structural morphogenesis and tissue development (adj. *P* < 1.69E-22), nucleosome assembly (adj. *P* < 7.00E-06), and lipid metabolic process (adj. *P* < 4.1E-06) (Fig. S2).

### Down-regulated and up-regulated transcripts for gastrointestinal disease states

**Figure 3:**
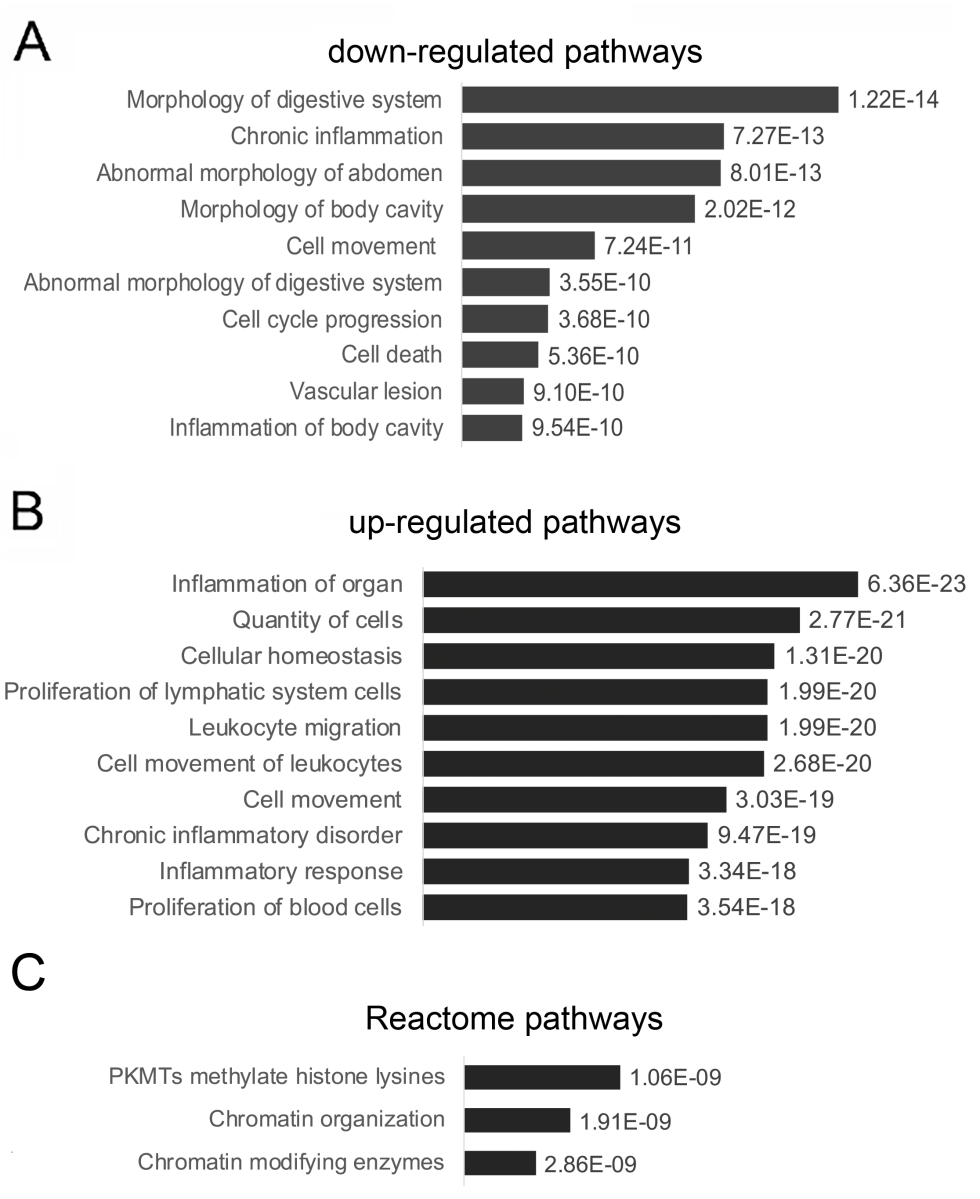
IPA analysis for biological pathway enrichment in colon epithelial cells in WAS rats. (A) The most significant down-regulated pathway as determined by IPA. (B) The most significant up-regulated pathway as determined by IPA. (C) Reactome pathway analysis of the second significantly upregulated gene transcripts.

Ingenuity Pathway Analysis™ (Qiagen, GmBH) was used to further analyze the RNA-seq datasets. To understand the networks involved in gastrointestinal disease following chronic psychological stress, we examined the output of the “diseases and functions” module. As shown in Fig. 3A, the down-regulated set of RNA transcripts in the WAS rat samples comprised two networks—a network that was labeled as “digestive system development and function” (*P*-values ranged from 1.29E-03–1.30E-09). The other smaller network of down-regulated genes, involved in gene expression and cell movement. Both networks were annotated with similar upstream regulators including estrogen (*P* = 5.80E-12), IGF1 (*P* = 1.47E-10) and EGF (*P* = 3.70E-10) as indicated by IPA.

IPA analysis further revealed two distinct functional networks with up-regulated gene sets. The first set included 3 functionally separated but interconnected pathways including “organismal injury and abnormalities” (*P* = 1E-65), “cellular movement, immune cell trafficking and connective tissue disorders” (*P* = 1E-52) and “tissue development” (*P* = 1E-24) (Fig. 3B). This set of up-regulated networks was significantly controlled by RELA (6.32E-17), TNF (2.68E-16) and IL4 (1.96E-15) as indicated by IPA. The most significant disease or disorder was “inflammation of organ” with *P*-value 6.36E-23 (Fig. 3B). In addition, a second distinct and separate network contained up-regulated transcripts that appeared to be involved in chromatin remodeling, based on trimethylation of histone 3 at lysine position 9 (H3K9me3) (Fig. 3C). This second significantly upregulated pathway is part of the human silencing complex (HUSH), which is responsible for repression of transcription.

### Chronic stress induced highly differentially expressed genes in a subpopulation of WAS rats

**Figure 4:**
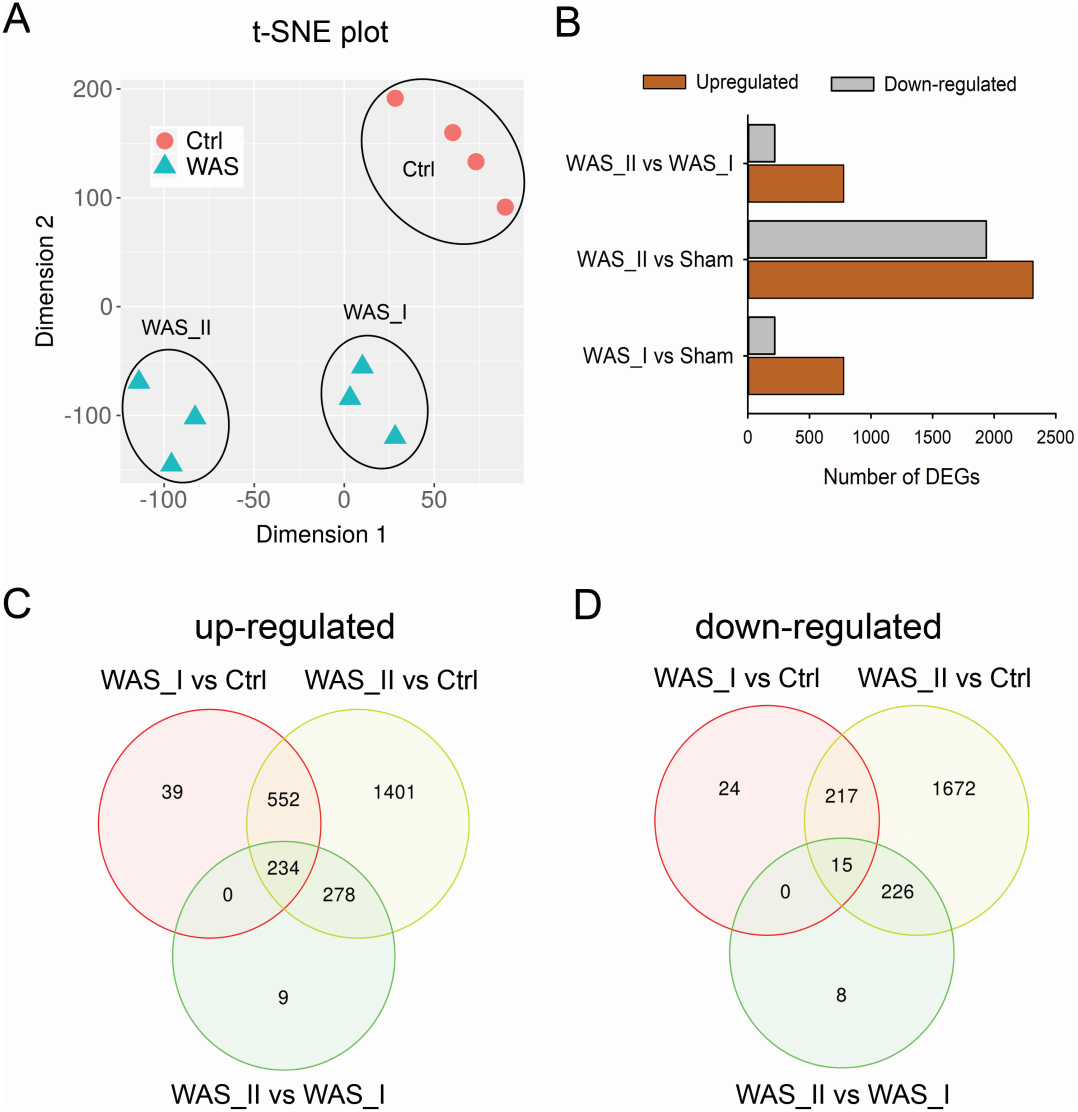
Subpopulation comparison of the differentially expressed genes (DEGs) in colon epithelial cells of WAS rats compared with controls. (A) t-SNE plot showed two distinct subpopulations of DEGs in WAS rat group. (B) Bar graph depicted the up-regulated and down-regulated DEGs in three comparisons: WAS_I *vs* Ctrl, WAS_II *vs* Ctrl, and WAS_II *vs* WAS_I. FDR < 0.01. (C) Venn diagram of up-regulated DEGs in WAS_I and WAS_II rats compared to the controls. (D) Venn diagram of down-regulated DEGs in WAS_I and WAS_II rats compared the controls.

We previously demonstrated that a subset of WAS rats developed visceral hypersensitivity which correlated with increased intestinal paracellular permeability (Creekmore et al., 2018), supporting a heterogeneous response to chronic stress in rats. Assessment using T-distributed stochastic neighbor embedding (t-SNE), a stochastic method to visualize large high dimensional datasets, demonstrated two subpopulations of WAS rats: WAS_I group (including WAS6, WAS7, WAS8) and WAS_II group (including WAS5, WAS9 and WAS10) (Fig. 4A). DEG analysis showed significantly higher numbers of upregulated and down-regulated genes in WAS_II group than WAS_I group when compared to the control group (Fig. 4B). Furthermore, the WAS_II group showed 778 upregulated gene transcripts and 218 down-regulated gene transcripts when compared to the WAS_I group (FDR < 0.01). Among the DEGs, 786 overlapping up-regulated and 326 overlapping down-regulated transcripts were detected between WAS_I and WAS_II groups when compared to the controls. There were 234 overlapping up-regulated and 15 overlapping down-regulated genes among the three comparisons (Figs. 4C & 4D).

**Figure 5:**
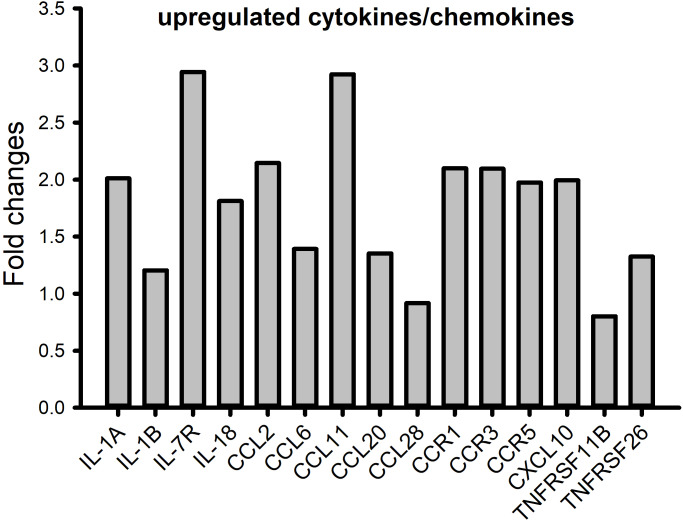
Significantly upregulated pro-inflammatory cytokine and chemokine genes in the WAS_II subpopulation compared to controls. Adj. *P* values < 0.01.

GSEA analysis revealed significant upregulated pathways related to nucleosome assembly/organization and immune response in the WAS_I group and immune response and chemokine signaling in the WAS_II group when compared with controls, respectively. The pathway related to immune response was also significantly upregulated in WAS_II group compared to WAS_I group (Table S5). Analysis of DEGs in the WAS_II subgroup rats showed a robust increase in the expression of pro-inflammatory cytokines and chemokines (with a stringent adjusted *P* < 0.001) including *IL1α*, *IL1β*, *IL7R, IL18, CCL2, CCL6, CCL11, CCL20, CCL28, CCR1, CCR5 and CXCL10*. Other inflammation-related transcripts were also significantly up-regulated such as *Tnfrsf11b and Tnfsf26* (Fig. 5).

### Changes in intestinal epithelial junctions following chronic psychological stress

**Figure 6:**
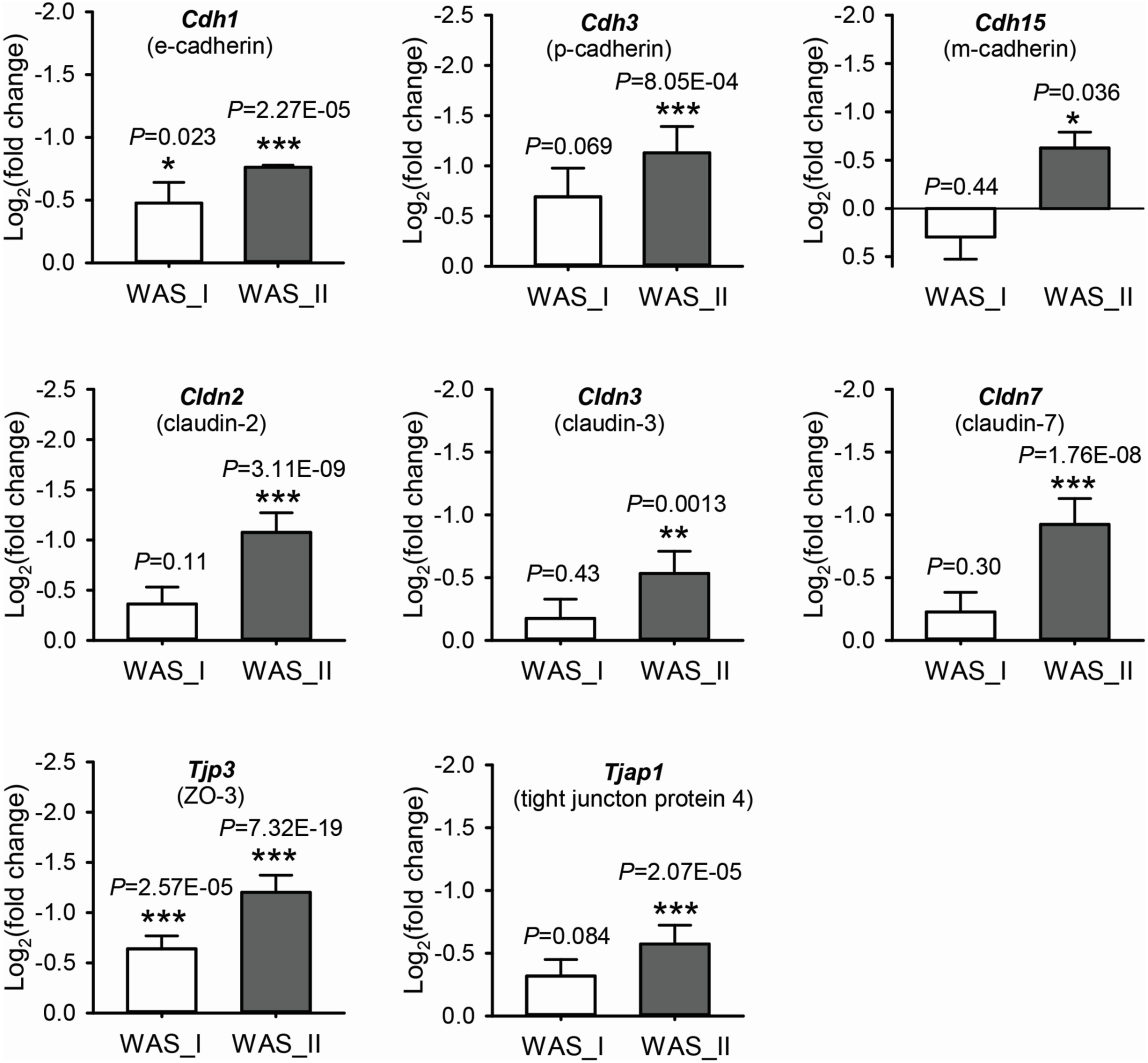
Differential alterations of down-regulated DEGs involving “tight junctions” in WAS subpopulations following chronic psychological stress compared to control rats. Data were expressed as mean ± SEM. *, adjusted *P* < 0.05 *vs* control; **, adjusted *P* < 0.01 *vs* control; ***, adjusted *P* < 0.001 *vs* control.

To analyze the potential difference in “tight junctions” between the WAS rat subpopulations in response to chronic stress, the fold changes and adjusted *P*-values (<0.05) of DEGs were compared regarding the down-regulation of adherens and intestinal epithelial tight junction genes. As shown in Fig. 6, the WAS_I subgroup rats showed significant decreases in *Cdh1* (e-cadherin) and *Tjp3* (tight junction protein 3 or ZO-3) compared to the control group. These two gene transcripts were further decreased and showed a much higher significance (adjusted *P* < 0.0001) in the WAS_II subgroup rats compared to the controls. Moreover, *Cdh3* (p-cadherin), *Cdh15* (m-cadherin), *Cldn2* (claudin-2), *Cldn3* (claudin-3), *Cldn7* (claudin-7) and *Tjap1* (tight junction associated protein 1 or tight junction protein 4) were also significantly decreased in WAS_II subgroup rats compared to the controls. These data indicate significant down-regulation in the expression of genes associated with epithelial cell barrier function (paracellular permeability) in the colon in the WAS_II subpopulation compared to the WAS_I subpopulation.

### Differential transcript usage in colon epithelial cells in chronic stress

**Figure 7:**
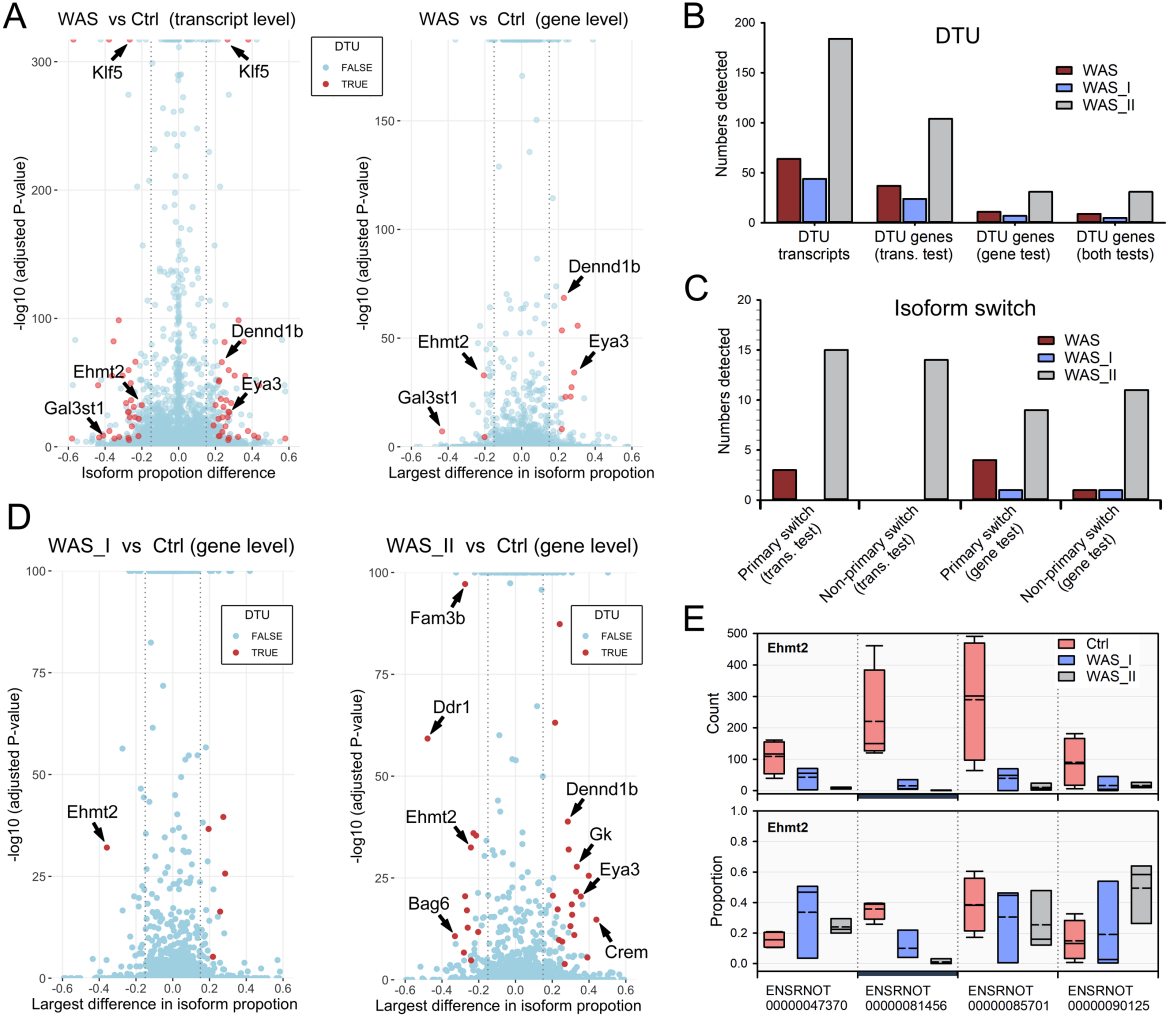
Differential transcript usage (DTU) in the stressed rats. (A) Transcript and gene-level tests revealed DTU events in the epithelial cells in the WAS rats. (B) Numbers of DTU transcripts and DTU genes in the WAS, WAS_I and WAS_II rat groups. (C) Numbers of isoform switch events in the WAS, WAS_I and WAS_II rat groups. (D) Comparison of DTU genes between the WAS_I and WAS_II rat groups. Arrows indicate DTU genes with identified isoform switching compared with the control group. (E) *Ehmt2* exhibited significant proportional isoform changes in the WAS_I and WAS_II subpopulations. Blue bar indicates the significantly changed *Ehmt2* isoform.

Using *salmon* counted transcript abundance and *RATs* R package, 64 events of differential transcript usage (DTU) were identified at the transcript-level in the WAS rat group, whereas the gene-level DTU test identified only 11 DTU genes (Fig. 7A). The following 9 genes were identified at the both transcript-level and gene-level: *Gal3st1*, *Ext2*, *Dym*, *Cnksr3*, *Dcun1d4*, *Eya3*, *Dennd1b*, *Elf1*, *Camsap2* and *Ehmt2*. Subpopulation analysis revealed a significant difference between the WAS_I and WAS_II rat groups. The WAS_I subpopulation showed 44 transcript-level DTU and 7 gene-level DTU, while the WAS_II subpopulation had 184 transcript-level DTU and 31 gene-level DTU (Fig. 7B). Examination of DTU events further showed isoform switches were more frequent in the WAS_II subpopulation than in the WAS_I subpopulation (Figs. 7C & 7D). In the WAS_I subpopulation, only *Ehmt2* exhibited primary and non-primary switching at the gene-level test. On the other hand, the WAS_II subpopulation exhibited 10 primary switching DTU and 14 non-primary switching DTU at the transcript-level test, and nine primary switching DTU and 11 non-primary switching DTU at the gene-level test. The following DTU genes exhibited both primary and non-primary switching in the WAS_II subpopulation in both tests: *Bag6*, *Crem*, *Ddr1*, *Dennd1b*, *Ehmt2*, *Eri3* and *Gk*. *Ehmt2*, known as a writer for H3K9 methylation, produces 4 transcripts and was found to be one of the common isoform switches in the whole group (WAS *vs* Ctrl) and subpopulation (WAS_I *vs* Ctrl and WAS_II *vs* Ctrl) comparisons. In normal colon epithelial cells, *Ehmt2* transcripts ENSRNOT00000081456 and ENSRNOT00000085701 were abundant. In chronic stress, the proportion of transcript ENSRNOT00000081456 was significantly lower and exhibited isoform switch in the WAS_I and WAS_II subpopulations (Fig. 7E) with a more apparent reduction in the WAS_II subpopulation.

### Super enhancers analysis in the colon epithelial cells

Super enhancers (SE), occupied by high densities of transcription factors and mediator complexes, are an important class of regulatory regions that appear to play critical roles in cell identity and regulation of cellular states in a variety of diseases (Lee & Young, 2013; Whyte et al., 2013). Genomic coordinates of SEs in human samples were analyzed using RefSeq (GRCh39/hg39) (Lee et al., 2022) and GeneHancer (Fishilevich et al., 2017) human gene annotations. Table S6 demonstrated relevant cell type-specific human SE targeting genes corresponding to the altered genes revealed by the RNA-seq using rat colon epithelial cells, including *Bag6*, *Ehmt2* and *Ddr1*. Although these three genes are located on different chromosomes in rats and humans, they share the same location in the genome and most likely are controlled by the same SE. In addition, the human homologs of the observed *Ehmt2* isoform switches (ENSRNOT00000081456 and ENSRNOT00000085701) in the rat colon epithelial cells between WAS subpopulations are also likely controlled by the same SE.

## Discussion

In this study we used RNA-seq to assess transcriptional changes within colon epithelial cells in rats following chronic intermittent water avoidance stress (WAS). Although DESeq2 and the D-GEX deep learning method yielded slightly different results, both methods identified the top up-regulation of *Atg16l1*, *Coq10b*, *Dcaf13*, *Nat2*, *Ptbp2*, *Rras2*, *Spink4*, and down-regulation of *Abat*, *Cited2*, *Cnnm2, Dab2ip*, *Plekhm1*, *Scd2*, and *Tab2* genes. Comparison of GWAS databases revealed that SNPs within the top up-regulated and down-regulated genes harbor gene variants that are significantly associated in humans with Crohn’s disease, chronic inflammatory disease, inflammatory bowel disease (IBD), ulcerative colitis, and depression. As a result, the most significantly altered genes revealed by bioinformatics methods may be used as potential biomarkers for chronic stress-induced intestinal barrier dysfunction and visceral hypersensitivity, given they are validated by other biochemical/molecular methodologies and in different chronic stress animal models.

Functional network enrichment analyses revealed that up-regulation of inflammatory/immune response was the most significantly altered biological process enriched with up-regulated genes including IL1*α*, IL1*β*, IL7R, IL18, *etc.* in the chronic WAS rat model. This is consistent with observations reported recently using the WAS model (Hattay et al., 2017; Xu et al., 2014) and in patients with IBS (Boyer et al., 2018; Bashashati, Moradi & Sarosiek, 2017; Chadwick et al., 2002). Patients suffering from post-traumatic stress disorder also have higher circulating levels of IL1*β*, IL6 and TNF-*α* in the peripheral blood (Von Kanel et al., 2007; Gill, Vythilingam & Page, 2008). Furthermore, our RNA-seq study revealed significant increases in chemokines and chemokine receptors including *CCL2*, *CCL6*, *CCL11*, *CCL20*, *CCL28*, *CCR1*, *CCR5* and *CXCL10*. Increased chemokines such as CCL2 and CCL20 at mucosal has recently been reported in IBS patients (Hayatbakhsh et al., 2019; Berg et al., 2020; Camilleri et al., 2014). The cytokine and chemokine expression has distinguished profiles between patients with IBS and IBD, suggesting that inflammatory mechanisms of the diseases are part of different spectrums (Moraes et al., 2020) yet may overlap in certain circumstances such as increased immune response and increased gut permeability (Spiller & Major, 2016). Elevation in cytokines is known to correlate with increased intestinal epithelial permeability *via* impaired expression and function of cell–cell tight junctions (Kakei et al., 2014; Amoozadeh et al., 2015), particularly in human GI disorders (Krug, Schulzke & M, 2014; Li et al., 2013). Consistently, our RNA-seq data demonstrated significant changes in the pathways related to digestive system development and structure morphology, including down-regulation of epithelial tight junction genes in WAS rats, especially in the WAS_II subpopulation. The loss of these paracellular junction proteins leads to the increase in paracellular permeability, sensitizes nerve terminals of afferent neurons and enhances visceral pain sensation. Our recent study supports the role of pro-inflammatory cytokines such as IL6 in down-regulation of tight junction proteins and increase in visceral hypersensitivity (Wiley et al., 2020).

Epigenetic regulation plays a significance role in numerous key physiological and pathophysiological processes (Denk & McMahon, 2012). Recent data supports both central and peripheral roles for epigenetic mechanisms in chronic stress-induced visceral hypersensitivity (Hong, Zheng & Wiley, 2015; Reul, 2014). In the current study, pathway analysis revealed a significantly up-regulated, histone H3K9 methylation-mediated nucleosome and chromatin remodeling in the colon epithelial cells in WAS animals. Methylation of H3K9, catalyzed by writers such as Ehmt1/Ehmt2, Setdb1 and Suv39h1/Suv39h2, is usually associated with silenced gene transcription and condensed chromatin (Hyun et al., 2017). Densely packed and transcriptionally silent heterochromatin is broadly enriched with stable H3K9me2/3 marks, which is also present at euchromatin to suppress active genes (Ninova, Toth & Aravin, 2019; Mozzetta et al., 2015; Saha & Muntean, 2021). In the WAS rats, Ehmt2 and Suv39h2 significantly increased and concurrently H3K9 demethylases such as Kdm3b, Kdm4a and Kdm4b decreased in the rat colon epithelial cells. Differential alterations of these enzymes can increase the H3K9 methylation status at epithelial tight junction gene promoters, culminating in suppression of gene transcription and increase in paracellular permeability (Wiley et al., 2020).

Heterogeneity is an important clinical feature of IBS symptoms (Linsalata et al., 2018). Besides the central feature of visceral hypersensitivity, animal models for IBS also demonstrate significant variability in reproducing the symptoms of IBS (Johnson et al., 2020; Moloney et al., 2015). Sub-group analysis of our RNA-seq data demonstrated two distinct subpopulations with significant differences in differentially expressed genes. Compared to WAS_I subpopulation, the WAS_II subpopulation displayed significantly higher number of altered genes and more significant changes in inflammatory response genes and biological pathways involving chemokine signaling. Correspondingly, the WAS_II subpopulation had a profound pattern of down-regulation in epithelial paracellular junction genes, suggesting that this subpopulation of animals were more vulnerable to chronic stress. Individual variability in paracellular permeability and visceral pain has been reported in the WAS rat model (Creekmore et al., 2018). The underlying mechanism(s) are unknown. Pre-exposure to early life stress such as maternal separation is likely to play a role, *via* epigenetic regulation, in the differential responses to chronic stress in the subpopulations of WAS animals (Fuentes & Christianson, 2018).

Stress-induced animal models for IBS have been widely used due to the reproducible outcome of increased intestinal paracellular permeability and visceral hypersensitivity (Zheng et al., 2013; Santos et al., 2000; Santos et al., 2001; Soderholm et al., 2002; Zareie et al., 2006). Corticotropin-releasing Factor (CRF), toll-like receptor 4 (TLR4) and serotonin 5-HT7 receptor (HTR7), associated with intestinal epithelial barrier dysfunction in the WAS model (Tache & Perdue, 2004; Arie et al., 2019; Zhu et al., 2019), were identified by our RNA-seq study although these genes were not among the list of the most significantly changed. This observation is consistent with clinical management of IBS patients using medicines that act peripherally on gut function targeting guanylate cyclase-c receptors, serotonin receptors, chloride channels, *etc* (Drossman et al., 2018). Moreover, our RNA-seq data revealed novel changes in genes responding to chronic stress. For example, Atg16l1, a part of a large protein complex that is necessary for autophagy, was significantly increased in the WAS rats. Alterations of Atg16l1 and its variants are associated with disease susceptibility of IBD (Murthy et al., 2014; Massey & Parkes, 2007), which links to psychological stress (Wang et al., 2019). Changes in Atg16l1 may regulate inflammatory cytokine response (Sorbara et al., 2013; Pott, Kabat & Maloy, 2018) to influence down-stream pathways such as morphology of epithelium including epithelial tight junctions. Another instance is the significantly down-regulated Stearoyl-CoA desaturase 2 (SCD2) gene. SCD2 catalyzes the formation of monounsaturated fatty acids from saturated fatty acids (Wang et al., 2005; Zhang, Yang & Shi, 2005). In IBD, translocation of gut microbial components resulting from increased paracellular permeability attenuates SCD activity and reduces fatty acid levels in red blood cells causing shortened life span (Kumar et al., 2020). Deletion of SCD from the intestinal epithelium promotes inflammation and tumorigenesis (Ducheix et al., 2018). In addition, the alterations of Mirlet7d (microRNA) and Cited2 (p300/CBP transcriptional coactivator) observed in WAS rats are likely involved in chromatin remodeling. Down-regulation of Cited2 can reduce histone acetylation and change chromatin accessibility (Liu, Chang & Chao, 2015). Overexpression of Mirlet7d inhibits H3K9 demethylation enzyme Kdm3a (Xu, Song & Lu, 2021), which subsequently may increase H3K9 methylation and lead to chromatin condensation. Mirlet7d has also been reported to inhibit anti-inflammatory cytokines such as IL10 and IL13 (Su et al., 2021), and therefore may play a role in the upregulation of inflammatory response in the WAS rats. Other top changed genes are mostly associated with cell progression and relevant signal transduction, including Dcaf13, Rras2, Abat, Alpk3, Dab2ip, Tab2 and Lrig3.

Isoform switches are implicated in many disease conditions. For example, differential exon usage (DTU) in ATPase is linked to major depression disorder (Pantazatos et al., 2017). Specific isoforms of trypsin and serotonin receptors are detected in tissues from IBS patients (Rolland-Fourcade et al., 2017; Wohlfarth et al., 2017). In the WAS rats, we observed differential transcript usage (DTU) for 9 genes including *Gal3st1*, *Ext2* and *Ehmt2*. Interestingly, the more vulnerable WAS_II subpopulation demonstrated significantly more DTU at both transcript and gene levels. Enriched functional network analysis revealed that DTU genes observed in the WAS_II subpopulation were identified to connect to transcriptional regulation network (*Ehmt2*, *Klf5*, *Ube2a*, *Elk4* and *Nacc1*), Serine/threonine/tyrosine protein kinase family (*Mark3*, *Cdc42bpb*, *Ephb2* and *Sik2*) and other signaling cascade (*Tab1*, *Ogt*, and *Ankrd17*). These genes are associated with cellular protein modification, regulation of cellular metabolic process, and regulation of cell morphogenesis as revealed by GO enrichment analysis. In post-infectious IBS patients, the expression of *Ephb2* is increased which promotes the potentiation of myenteric nerves and enhances pain perception (Zhang et al., 2019). Currently, understanding about the functional differences in expressed isoforms is limited to a small portion of proteins. Our findings of differential exon usage and isoform switches in colon epithelial cells in the WAS model may contribute to the understanding of the complex phenotypes and heterogeneity of IBS. The discovery of biomarkers based on differential exon usage has the potential to predict phenotypes not accurately predicted by general gene expression profiles (Sadeque et al., 2012).

Cell type-specific super enhancers embraces the master transcription factors and are sensitive to extrinsic stimuli in the specific type of cells they are expressed. Super enhancers enriched for disease-associated SNPs in genome wide association studies (GWAS) may thus serve as genome substrates for cell- and tissue-specific disorders (Whyte et al., 2013; Grosveld, Van Staalduinen & Stadhouders, 2021; Hnisz et al., 2013). In humans, 16 different single nucleotide variants (SNPs) located within the *Atg16l1* gene and in an intergenic region between the *Atg16l1* and *Scarna5* genes have been associated with inflammatory bowel disease, ulcerative colitis, and Crohn’s disease (Buniello et al., 2019; Liu et al., 2015; Franke et al., 2010; Julia et al., 2013). In humans these genes are regulated by 3 shared super-enhancers (Table S6) (Khan & Zhang, 2016). It is tempting to speculate that increases in the expression of these genes in the rat WAS model may be due to dysregulation of this control system, providing a model for the genesis of stress-related bowel disorders. Similarly, the DTU genes that exhibited both primary and non-primary switching in the WAS_II subpopulation included the *Bag6*, *Ddr1*, and *Ehmt2* genes located close together along with the long non-coding regulatory RNAs *ENSRNOT0000081456* and *ENSRNOT0000085701* on chromosome 20 in the rat (Haeussler et al., 2019) and their human homologs are located together on chromosome 6 and share enhancer and super-enhancer regulation in Homo sapiens. This suggests that defects in the cis regulation of gene expression may be responsible, in part, for some of the differences that we observed in this study.

The limitation of this study is the modest sample sizes. However, this does not diminish the validity of the observations which are based on rigorous bioinformatics analyses. We expect that large samples sizes of RNA-seq analysis followed by q-PCR and protein quantitation in future studies will help confirm and validate the observations from the WAS animal model. The colon crypt contained multiple type of cells and heterogeneity information cannot be obtained by regular RNA-seq study. Single cell sequencing analysis using the sorted epithelial cells (Zhang et al., 2020; Smillie et al., 2019; Chen et al., 2021) will also help elucidate the distinct changes in expression and function of specific types of cells along the crypt axis in regulation of intestinal barrier function and visceral pain perception in chronic stress.

## Conclusions

In summary, our RNA-seq analysis using the colon epithelial cells from the chronic stress rat model revealed significantly altered gene transcripts and biological pathways relevant to inflammation/immune response, tissue morphogenesis and development, and nucleosome/chromatin assembly in the subpopulation of stressed rats. These findings support that chronic stress is associated with increased levels of cytokines and chemokines, their downstream signaling pathways coupled to dysregulation of intestinal cell development and function. Epigenetic regulation of chromatin remodeling likely plays a prominent role in this process. As “proof of concept”, this study has certain limitations. For example, due to the complexity of the colon epithelial cells profiled in this study, this RNA-seq dataset cannot be used to classify the differential transcript changes in the specific cell types in the colon crypt that include stem cells, proliferating cells and differentiated cells along the crypt axis. Single-cell RNA-seq and single-nuclei RNA-seq will be the complementary tool to decode the heterogeneity in this complex tissue by generating transcriptomic profiles of the individual cell in future studies.

## Supplemental Information

10.7717/peerj.13287/supp-1 Supplemental Information 1 Supplementary Figures

10.7717/peerj.13287/supp-2 Supplemental Information 2 Supplementary Tables

10.7717/peerj.13287/supp-3 Supplemental Information 3 Supplementary Methods

10.7717/peerj.13287/supp-4 Supplemental Information 4 Raw gene counts from RNA-seq study

10.7717/peerj.13287/supp-5 Supplemental Information 5 ARRIVE 2.0 checklist

## Abbreviations

ChIP: Chromatin Immunoprecipitation
DEG: differentially expressed genes
DTU: differential transcript usage
IBS: Irritable Bowel Syndrome
IPA: Ingenuity Pathway Analysis
SE: super enhancer
WAS: water avoidance stress

## Additional Information and Declarations

### DNA Deposition

The following information was supplied regarding the deposition of DNA sequences: The raw RNA-seq data has been deposited to NCBI database with accession number PRJNA764901.

### Data Availability

The following information was supplied regarding data availability: The raw measurements are available in the Supplementary File.
