# Supplementary material for: Chronic psychological stress alters gene expression in rat colon epithelial cells promoting chromatin remodeling, barrier dysfunction and inflammation
Source: PeerJ. 2022 Apr 29;10:e13287. doi: 10.7717/peerj.13287 (PMC9059753; doi:10.7717/peerj.13287)

**Supplementary Figure 1: Schematic workflow of next-generation RNA-seq and bioinformatics analysis.** After adaptation for 7 days in the animal facility, young-adult male rats were subjected to intermittent (1-hr daily for 10-days) water avoidance stress (WAS) or sham stress. Sections of distal colon were removed, epithelial cells enriched and total RNA extracted for cDNA library preparation. Sequencing was performed on Illumina HiSeq 4000 platform and the generated raw reads were subjected to bioinformatics analysis including quality control, alignment/annotation, differential expression analysis, GO enrichment analysis, KEGG, Ingenuity Pathway Analysis (IPA) and subpopulation analysis.

**
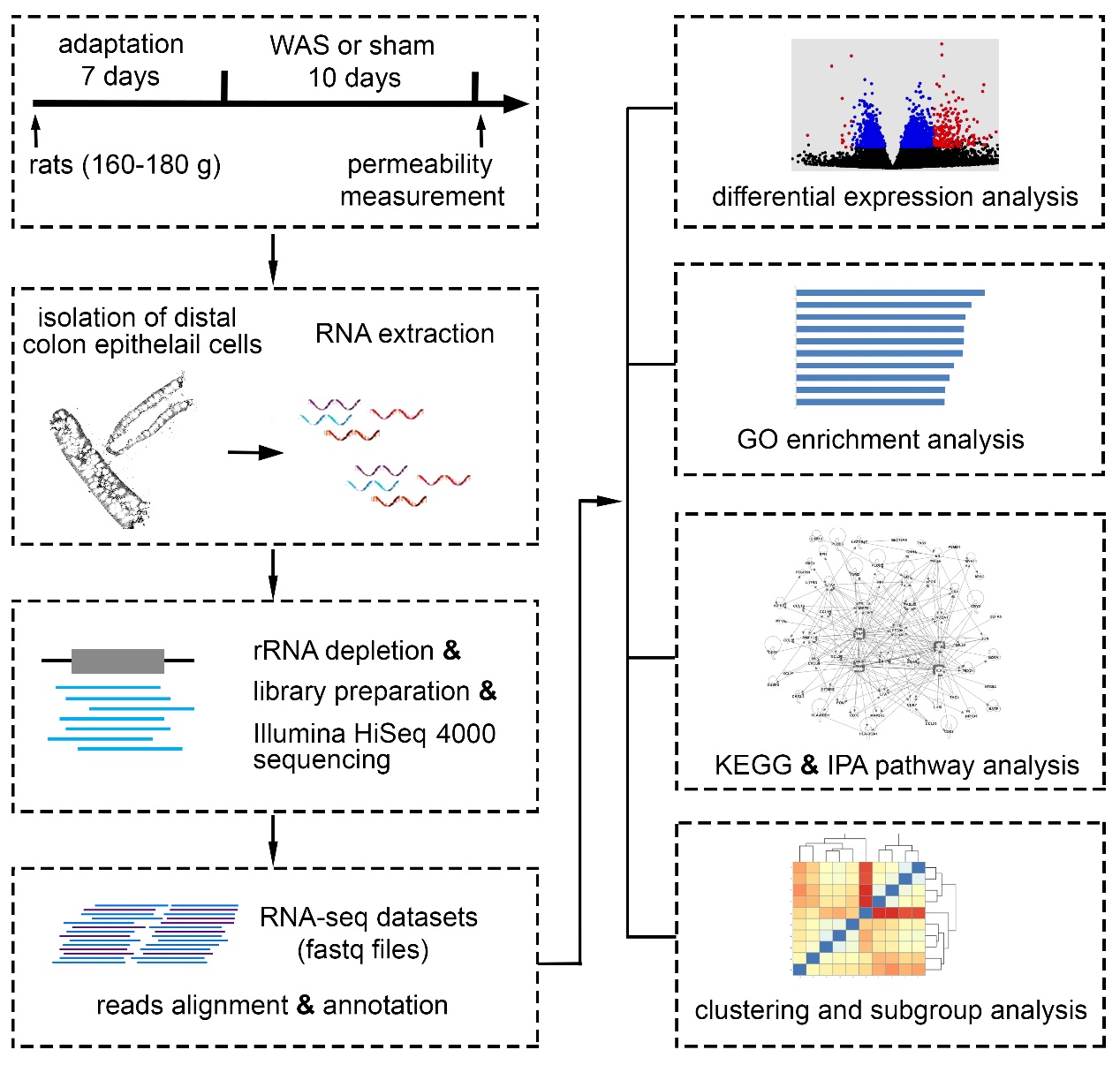
**

**Supplementary Figure 2: K-means clustering of top 2000 differentially expressed genes in colon epithelial cells in the WAS rats.** The number of clusters was determined by elbow method and t-SNE plot. Enriched GO biological process terms were shown in Cluster A, B, C and D.


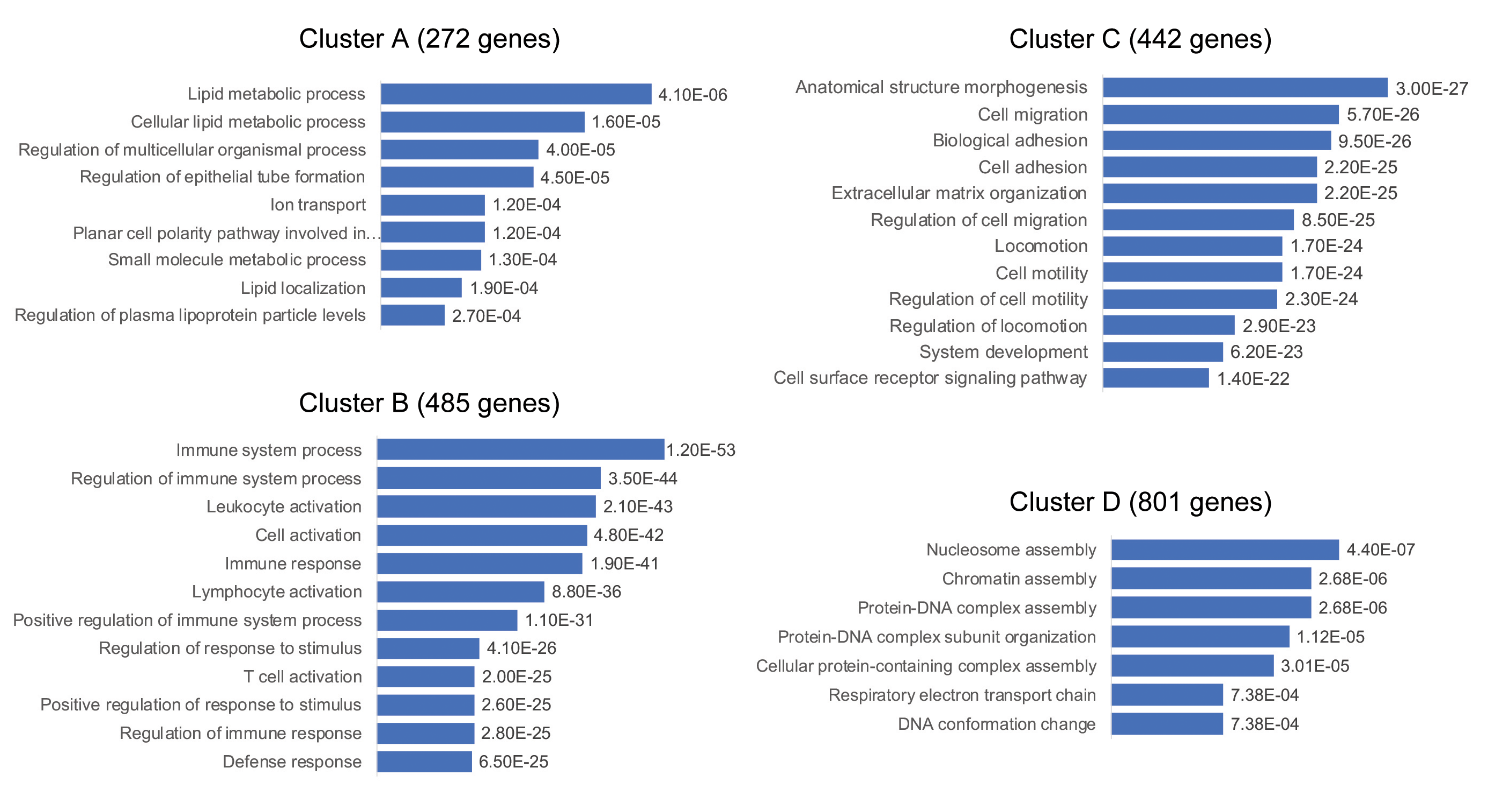

Supplement: Supplemental Information 1 [file peerj-10-13287-s001.docx]
