# Supplementary material for: Chronic psychological stress alters gene expression in rat colon epithelial cells promoting chromatin remodeling, barrier dysfunction and inflammation
Source: PeerJ. 2022 Apr 29;10:e13287. doi: 10.7717/peerj.13287 (PMC9059753; doi:10.7717/peerj.13287)
Supplement: Supplemental Information 2 [file peerj-10-13287-s002.docx]

**Supplementary Table 1. Summary of RNA-seq reads alignment to the rat genome.** **, *P* < 0.01 between WAS and control (Ctrl) rat groups.

| **Treatment** | **Raw reads** | **Alignment** | | | |
| --- | --- | --- | --- | --- | --- |
|  |  | **Coding**  **(%)** | **UTR**  **(%)** | **Intron**  **(%)** | **Intergenic**  **(%)** |
| **Ctrl** | 1.50E+8 | 29.34±1.60 | 17.83±1.78 | 26.55±1.30 | 26.28±0.33 |
| **WAS** | 1.31E+8 | 22.47±1.12** | 26.45±1.30** | 24.25±3.07 | 26.83±1.10 |

**Supplementary Table 2. Summary of the top 10 up-regulated and down-regulated genes in the colon epithelial cells in WAS rats using conventional DESeq2 method.**

| **Gene** | **Gene description** | **Log_2_ Fold change** | **Raw *P* value** | **Adjusted *P* value** |
| --- | --- | --- | --- | --- |
| **Upregulated** | | | | |
| *Spink4* | serine peptidase inhibitor, Kazal type 4 | 5.02 | 1.93E-12 | 1.41E-09 |
| *Mirlet7d* | microRNA let-7d | 2.7 | 1.26E-17 | 8.84E-14 |
| *Atg16l1* | Autophagy-related 16-like 1 | 2.35 | 1.58E-17 | 8.84E-14 |
| *Nat2* | N-acetyltransferase 2 | 1.8 | 7.39E-12 | 3.14E-09 |
| *Coq10b* | coenzyme Q10B | 1.57 | 1.59E-12 | 1.21E-09 |
| *Dcaf13* | DDB1 and CUL4 associated factor 13 | 1.49 | 8.07E-12 | 3.14E-09 |
| *Fancb* | FA complementation group B | 1.43 | 1.26E-11 | 4.14E-09 |
| *Rpp40* | ribonuclease P/MRP subunit p40 | 1.34 | 2.97E-12 | 1.78E-09 |
| *Ptbp2* | polypyrimidine tract binding protein 2 | 1.23 | 6.43E-13 | 5.37E-10 |
| *Rras2* | RAS related 2 | 0.88 | 1.55E-15 | 4.32E-12 |
| **Down-regulated** | | | | |
| *Scd2* | stearoyl-Coenzyme A desaturase 2 | -2.01 | 4.67E-19 | 7.82E-15 |
| *Abat* | 4-aminobutyrate aminotransferase | -1.24 | 3.81E-10 | 5.18E-09 |
| *Alpk3* | alpha-kinase 3 | -1.15 | 1.47E-09 | 6.50E-07 |
| *Dab2ip* | DAB2 interacting protein | -0.97 | 2.18E-12 | 1.46E-09 |
| *Tex2* | testis expressed 2 | -0.95 | 1.10E-11 | 3.92E-09 |
| *Cnnm2* | cyclin and CBS domain divalent metal cation transport mediator 2 | -0.93 | 3.23E-10 | 4.74E-08 |
| *Tab2* | TGF-beta activated kinase 1/MAP3K7 binding protein 2 | -0.92 | 8.07E-14 | 1.23E-10 |
| *Lrig3* | Leucine Rich Repeats and Immunoglobulin Like Domains 3 | -0.82 | 6.61E-11 | 1.48E-08 |
| *Plekhm1* | pleckstrin homology and RUN domain containing M1 | -0.71 | 5.98E-11 | 1.35E-08 |
| *Cited2* | Cbp/p300-interacting transactivator | -0.78 | 2.14E-08 | 1.27E-06 |

**Supplementary Table 3. Summary of the top 10 up-regulated and down-regulated genes in the colon epithelial cells in WAS rats using a deep learning method.**

| **Gene** | **Gene Name** | **log2 fold Change** | **t-statistic** | ***p*-value** |
| --- | --- | --- | --- | --- |
| **Upregulated** | | | | |
| *Spink4* | Serine peptidase inhibitor, Kazal type 4 | 7.34 | 8.01 | 0.0002 |
| *Atg16L1* | Autophagy-related 16-like 1 | 6.24 | 4.5 | 0.00008 |
| *Nat2* | N-acetyltransferase 2 | 6.07 | 4.79 | 0.0003 |
| *Coq10b* | Coenzyme Q10B | 5.45 | 5.08 | 0.00006 |
| *Dcaf13* | DDB1 and CUL4 associated factor 13 | 5.03 | 4.27 | 0.0001 |
| *Fancb* | FA complementation group B | 4.96 | 4.21 | 0.001 |
| *Ptbp2* | Polypyrimidine tract binding protein 2 | 3.99 | 6.28 | 0.000002 |
| *Rras2* | RAS related 2 | 3.99 | 4 | 0.000012 |
| *Scarna5* | Small Cajal Body-Specific RNA 5 | 3.97 | 4.5 | 0.0008 |
| *Nod2* | Nucleotide Binding Oligomerization Domain Containing 2 | 3.97 | 4.14 | 0.0011 |
| **Downregulated** | | | | |
| *Plekhm1* | Pleckstrin homology and RUN domain containing M1 | -2 | -5.17 | 0.00006 |
| *Lrig3* | Leucine Rich Repeats and Immunoglobulin Like Domains 3 | -2.02 | -6.92 | 0.0003 |
| *Cited2* | Cbp/p300-interacting transactivator | -2.2 | -7 | 0.00007 |
| *Lrig3* | Leucine Rich Repeats and Immunoglobulin Like Domains 3 | -2.25 | -5.59 | 0.0003 |
| *Tab2* | TGF-beta activated kinase 1/MAP3K7 binding protein 2 | -2.5 | -7.26 | 0.00002 |
| *Cnnm2* | Cyclin and CBS domain divalent metal cation transport mediator 2 | -2.86 | -6.83 | 0.0003 |
| *Dab2ip* | DAB2 interacting protein | -2.9 | -6.2 | 0.00004 |
| *Abat* | 4-aminobutyrate aminotransferase | -2.95 | -3.56 | 0.0002 |
| *Alpk3* | Alpha kinase 3 | -2.96 | -4.48 | 0.008 |
| *Scd2* | Stearoyl-Coenzyme A desaturase 2 | -3 | -6.14 | 0.0004 |

**Supplementary Table 4**. **Examples of human homologous/orthologous genes, single nucleotide polymorphisms (SNPs) from genome wide association studies (GWAS), variant types and trait associations.** Citations to GWAS indicated by PubMed ID number.

| **Human analog** | **SNP** | **Disease trait** | ***p*-value** | **References [PubMed ID number]** |
| --- | --- | --- | --- | --- |
| *ATG16L1* | rs6738490 | Crohn’s disease | 4E-78 | 26192919 |
| *ATG16L1* | rs12994997 | Crohn’s disease | 4E-70 | 23128233 |

| *ATG16L1* | rs35300242 | Chronic inflammatory disease | 5E-67 | 26970047 |
| --- | --- | --- | --- | --- |
| *ATG16L1* | rs6752107 | Crohn’s disease | 2E-61 | 28067908 |
| *ATG16L1* | rs12994997 | Inflammatory bowel disease | 3E-41 | 26192919 |
| *SCARNA5,*  *ATG16L1* | rs3792109 | Crohn’s disease | 7E-41 | 21102463 |
| *ATG16L1* | rs3828309 | Crohn’s disease | 2E-32 | 18587394 |
| *ATG16L1* | rs6752107 | Inflammatory bowel disease | 3E-28 | 28067908 |
| *ATG16L1* | rs3792108 | Ulcerative colitis | 4E-17 | 33686288 |
| *SCARNA5,*  *ATG16L1* | rs3792109 | Rheumatoid arthritis, Crohn’s disease | 2E-16 | 33686288 |
| *ATG16L1* | rs10210302 | Crohn’s disease | 5E-14 | 17554300 |
| *ATG16L1* | rs2241880 | Crohn’s disease | 1E-13 | 17435756 |
| *ATG16L1* | rs2241880 | Crohn’s disease | 1E-12 | 22412388 |
| *SCARNA5,*  *ATG16L1* | rs3792109 | Crohn’s disease | 5E-09 | 22936669 |
| *PTBP2* | rs720090 | Anorexia nervosa, unipolar depression | 2E-08 | 33686288 |
| *CNNM2* | rs4919695 | Feeling nervous | 1E-13 | 29500382 |
| *CNNM2* | rs1890184 | Anxiety | 2E-13 | 29942085 |
| *CNNM2* | rs4919695 | Worry | 2E-13 | 29500382 |
| *CNNM2* | rs7921574 | Feeling nervous | 3E-13 | 29500382 |
| *CNNM2* | rs94035 | Worry | 8E-13 | 29500382 |
| *CNNM2* | rs11191548 | Major depressive disorder | 8E-11 | 34159505 |
| *CNNM2* | rs11191499 | Neuropsychiatric disorders, including major depressive disorder | 1E-09 | 33479212 |
| *CNNM2* | rs11191499 | Major depressive disorder | 1E-09 | 33479212 |
| *CNNM2* | rs7914558 | Neuropsychiatric disorders, including major depressive disorder | 2E-09 | 23453885 |
| *CNNM2* | rs3902934 | Anxiety | 2E-08 | 30867560 |
| *SPINK4/*  *BGALT1-AS1* | rs830407 | Major depressive disorder | 4E-07 | 29662059 |
| *SPINK4* | rs28447319 | Influenza A (H1N1) infection | 1E-14 | 26379185 |
| *TAB2* | rs7758080 | Crohn’s disease | 7E-09 | 26192919 |
| *TAB2* | rs7758080 | Inflammatory bowel disease | 1E-07 | 26192919 |
| *NOD2, CYLD-AS1* | rs5743293 | Chronic inflammatory disease | 1E-320 | 26974007 |
| *CYLD-AS1, NOD2* | rs2066847 | Crohn’s disease | 6E-209 | 23128233 |
| *NOD2* | rs2066844 | Crohn’s disease | 6E-99 | 28067908 |
| *NOD2* | rs2066845 | Ulcerative colitis, Crohn’s disease | 6E-94 | 26974007 |
| *NOD2* | rs2076756 | Crohn’s disease | 4E-69 | 21102463 |
| *NOD2* | rs2066844 | Mouth ulcers | 3E-40 | 30837455 |
| *NOD2* | rs2066844 | Inflammatory bowel disease | 1E-38 | 28067908 |
| *NOD2* | rs2076756 | Crohn’s disease | 1E-37 | 22412388 |
| *NOD2* | rs2076756 | Ulcerative colitis, Crohn’s disease | 5E-29 | 33686288 |
| *CYLD-AS1, NOD2* | rs2066847 | Crohn’s disease | 3E-24 | 18587394 |
| *NOD2* | rs2076756 | Crohn’s disease | 1E-21 | 17684544 |
| *NOD2* | rs5743289 | Crohn’s disease | 6E-17 | 17804789 |
| *CYLD-AS1, NOD2* | rs2066847 | Ankylosing spondylitis, ulcerative colitis, Crohn’s disease, psoriasis | 1E-16 | 26974007 |
| *CYLD-AS1, NOD2* | rs2066847 | Crohn’s disease | 2E-15 | 20570966 |
| *NOD2* | rs2076756 | Crohn’s disease | 7E-14 | 17435756 |
| *NOD2* | rs2076756 | Rheumatoid arthritis, Crohn’s disease | 5E-13 | 33686288 |
| *NOD2* | rs17221417 | Crohn’s disease | 4E-11 | 17554300 |
| *NOD2* | rs2066844 | Asthma (childhood onset) | 1E-10 | 30929738 |
| *NOD2* | rs2076756 | Crohn’s disease | 3E-10 | 22936669 |
| *NOD2* | rs5743289 | Inflammatory bowel disease | 4E-10 | 18758464 |
| *NOD2* | rs2076756 | Inflammatory bowel disease | 5E-10 | 17068223 |
| *CYLD-AS1, NOD2* | rs2066847 | Inflammatory bowel disease | 2E-09 | 36608531 |

**Supplementary Table 5. Summary of the top 10 up-regulated biological pathways in the colon epithelial cells in WAS subpopulations using preranked GSEA method.**

**Supplementary Table 6**. **Examples of human homologous/orthologous genes, single nucleotide polymorphisms (SNPs) from genome wide association studies (GWAS), variant types, trait associations, and tissue specificity indicated by super-enhancer status of four of the most significantly upregulated transcripts in the WAS rat model.** TAD: Topologically associating domain. Citations to GWAS indicated by PubMed ID number.

| **Transcript** | **Gene definition** | **Examples of human GWAS SNPs** | **Variant type** | **Trait association** | ***P*-value;**  **OR [CI]** | **PubMed ID** | **Tissue-specific super-enhancer(s)** | **Super-enhancer targets** |
| --- | --- | --- | --- | --- | --- | --- | --- | --- |
| *ATG16L1* | Autophagy Related 16 Like 1 | rs6738490 | Intragenic enhancer;  *ATG16L1* | Crohn’s disease | 4E-70;  1.223 [1.193-1.274] | 23128233 | SE_18683  (CD+4 CD25- 1l17- PMA Th primary cells);  SE_15254  (CD4 memory primary cells);  SE_22578  (CD8 primary cells) | *ATG1611; SCARNA5; SCARNA6; ENSG00000271639; AYO77737* |
|  |  | rs35300242 | Intragenic enhancer;  *ATG16L1* | Chronic inflammatory diseases (ankylosing spondylitis, Crohn’s disease, psoriasis, primary sclerosing cholangitis, ulcerative colitis- (pleiotropy)) | 5E-67 | 26974007 |  |  |
| *NOD2* | Nucleotide Binding Oligomerization Domain Containing 2 | rs5743293 | Frameshift;  *NOD2* | Chronic inflammatory diseases (ankylosing spondylitis, Crohn’s disease, psoriasis, primary sclerosing cholangitis, ulcerative colitis- (pleiotropy)) | 1E-320 | 26974007 | SE_19501  (CD+4 CD25- IL17+ PMA Th17 primary cells);  SE_26957  (Esophagus);  SE_17258  (CD+4 CD25int CD127+ Tmem primary cells);  SE_09558  (CD+14 monocytes);  SE_16843  (CD4 naïve primary cells);  SE_22990  (CD8 primary cells);  SE_18724  (CD+4 CD25- IL17+ PMA Th17 primary cells);  SE_17967  (CD+4 CD25- CD45RO+ primary memory cells) | *NOD2; SNX20; HNRNPA1P48; CYLD; ADCY7; NONHSAG019425.2;*  *pIR-51401* |
|  |  | rs2066847-T | Frameshift;  *NOD2* | Crohn’s disease | 6E-209;  3.103 | 23128233 |  |  |
|  |  | rs2066844 | Missense SNP located on TAD boundary;  *NOD2_ NONHSAG019425.2* | Inflammatory bowel disease | 1E-38 | 28067908 |  |  |
| *SCARNA5* | Small Cajal Body-specific RNA 5 | rs3792109-A | Intergenic enhancer;  *ATG16L1_SCARNA5* | Crohn’s disease | 7E-41;  1.34 [1.29-1.40] | 21102463 | SE_18683  (CD+4 CD25- 1l17- PMA Th primary cells);  SE_15254  (CD4 memory primary cells);  SE_22578  (CD8 primary cells) | *ATG1611; SCARNA5; SCARNA6; ENSG00000271639; AYO77737* |
|  |  | rs3792109-A | Intergenic enhancer;  *ATG16L1_SCARNA5* | Crohn’s disease | 5E-09;  1.38 | 22936669 |  |  |
| *SPINK4* | Serine Peptidase Inhibitor Kazal Type 4 | rs28447319 | Promoter;  *SPINK4* | Severe influenza A (H1N1) infection | 1E-14;  21.08 [7.017-62.55] | 26379185 | SE_24282, SE_23691  (Colon crypt);  SE_15181  (CD4 primary cells);  SE_20411  (CD56 primary cells);  SE_13983  (CD34 primary cells):  SE_22826  (CD8 primary cells);  SE_11537  (CD+20 B cells) | *SPINK4; B4GALT1; BAG1; CHMP5; SMU1; NFX1; B4GALT1-AS1; RNU4ATAC15P;*  *lnc-B4GALT1-2;*  *lnc-BAG1-1,2; RF00017-750-1; RF00017-7499* |
