# Supplementary material for: Chronic psychological stress alters gene expression in rat colon epithelial cells promoting chromatin remodeling, barrier dysfunction and inflammation
Source: PeerJ. 2022 Apr 29;10:e13287. doi: 10.7717/peerj.13287 (PMC9059753; doi:10.7717/peerj.13287)
Supplement: Supplemental Information 3 [file peerj-10-13287-s003.docx]

**Supplementary Methods**

**Differential gene expression analysis using deep learning**

Independent analysis performed in a blinded manner using deep learning to quantify differentially expressed genes (DEGs) validated the results of the other methods, although it was not able to detect microRNA, other short RNA species, or alternative splicing. A modification of the D-GEX method was used for high-throughput RNA-Seq data analysis.^1^  D-GEX is a multi-task multi-layer feedforward neural network containing an input layer, multiple hidden layers, and one output layer. The method was modified and applied as has been previously described.^2^ The D-GEX software is available at <https://github.com/uci-cbcl/D-GEX>. Analysis of differential transcript expression was performed independently using this method in parallel and analyzed in a blinded manner as previously described.^2^

**Analysis of super enhancers, single nucleotide polymorphisms (SNPs) and their associated genes**

Candidate super-enhancers (SEs) were identified using orthogonal results from other studies, including positional binding of the transcription factors Ctcf, Rad21, Smc3, Brd4, Brd4, Ep300, RNA polymerase 2A, and members of the mediator complex including Med1, co-localization with H3k27ac, and composition of multiple enhancers spanning adjacent topologically associating domains (TADs) containing lineage-specific transcription factors that determine cell type. In the rat, these are considered “candidate super-enhancers” as there is not yet sufficient evidence from genome editing methods such as Crispr-Cas for validation of super-enhancer function.

**Reference:**

1. Chen Y, Li Y, Narayan R, et al. Gene expression inference with deep learning. Bioinformatics 2016;32:1832-9.

2. Higgins GA, Georgoff P, Nikolian V, et al. Network Reconstruction Reveals that Valproic Acid Activates Neurogenic Transcriptional Programs in Adult Brain Following Traumatic Injury. Pharm Res 2017;34:1658-1672.
